# First report of HIV-related oral manifestations in Mali

**Published:** 2012-01-31

**Authors:** Irene Tamí-Maury, Yaya Ibrahim Coulibaly, Souare Salimata Cissoko, Sounkalo Dao, Sibylle Kristensen

**Affiliations:** 1 University of Alabama at Birmingham, Department of Epidemiology, Birmingham, United States (Currently at The University of Texas MD Anderson Cancer Center, Houston, United States); 2 Faculté de Médecine, de Pharmacie et d’Odonto-Stomatologie, Bamako, Mali; 3 Pharmacie/Centre Hospitalier Universitaire Gabriel Touré, Bamako, Mali; 4 Maladies Infectieuses et Tropicales/Centre Hospitalier Universitaire Point G, Bamako, Mali; 5 One Heart World-Wide, San Francisco, United States

**Keywords:** Oral manifestations, HIV, CD4 count, prevalence, ART

## Abstract

**Introduction:**

In 2004, the sudden availability of free antiretroviral therapy (ART in Mali, within the context of an already overburdened health care system created gaps in individual patient quality of care. The objective of this study was to determine the prevalence of HIV-related oral manifestations (OM) during the first month of ART therapy in a Malian health facility.

**Methods:**

Medical records of adult patients who initiated ART regimens at the Gabriel Touré Hospital, Mali (2001 to 2008) were randomly identified. Multiple logistic regression models were used to evaluate the relationship between the presence of OM during the first month of ART and selected variables, including CD4 counts and WHO clinical staging at ART initiation.

**Results:**

Out of 205 patients on ART (mean age 39 ± 10 years), 71.0% were females and 36.1% had no formal education. 40.6% were in WHO clinical stage III. OM prevalence during the first month of HIV care was 31.4%, being oral candidiasis the commonest lesion. 73.2% and 82.5% of the patients with OM had CD4 count < 200 cells/mm3 and were classified as WHO clinical stage III or IV. WHO clinical stage III and VI patients had 5.4-fold increased odds of having any OM (both p< 0.01) when controlling for age, ethnicity, gender, marital status, and CD4 counts.

**Conclusion:**

OM detected in people with low CD4 count and WHO clinical stage III and IV at ART initiation suggested that they were very immune-compromised when initiating HIV care. Early identification of OM could improve the quality of care and guarantee the benefits of ART.

## Introduction

While West Africa has been less affected than Sub-Saharan Africa by the HIV/AIDS epidemic, HIV prevalence is rising in some West African countries. In 2006, HIV prevalence estimates exceeded 7% in Côte d′Ivoire, a country neighboring Mali [1]. In Mali, the most recent data collected during a 2006 Demographic and Health Survey, indicate a possible decline in the epidemic [2]. National adult HIV prevalence was estimated at 1.2%, as compared to the 1.7% prevalence that a similar survey reported in 2001. However, mortality could be a contributing factor to the decline in prevalence. Rates of HIV infection are higher in Malian women than in men, particularly among pregnant women 25–29 years of age, among whom prevalence is almost 4%. Bamako, the capital city, has a higher prevalence of HIV/AIDS as compared to other Malian cities [3]. In January 2004, Mali declared free access to antiretroviral therapy (ART) [4]. By the end of 2006 the percentage of HIV-infected women and men receiving ART in the country was 37% [1]. The emphasis on high uptake of antiretroviral therapy in a short period of time within the context of an already overburdened health care system, especially in Bamako, has left gaps in the individual patient quality of care, including oral care. In 2003, a retrospective study carried out over three months (February to April 2002) at the Gabriel Touré Hospital in Bamako, showed that from a sample of 691 patients seeking care at the Ear, Nose and Throat Service, 19 patients were HIV positive. These results highlight the need to improve the screening of oro-pharyngo-laryngology manifestations (OM) of HIV/AIDS infection, manifestations that may not always be easily identified but that can definitely hamper the quality of life of a person living with HIV/AIDS (PLWHA) [5–7]. The presence of OM usually creates oral discomfort, which can result in compromised nutrition [8,9] and ART adherence [10]. Based on this fact, we conducted a study to determine the prevalence of OM in a population initiating ART therapy in Bamako, Mali.

## Methods

### Study population and data collection methods

Medical records were identified through a computer-aided random selection system from a list of 1,497 medical records of HIV adult patients (21 years old and older) registered from December 2001 to December 2008 in the Pharmacy of the Gabriel Touré Hospital. The study sample consisted of 205 patients who had initiated ART regimens at Gabriel Touré Hospital between December 2001 and December 2008. Data from the medical charts were recorded on data abstraction forms that were further reviewed. Only those OM reported during the first month of ART therapy initiation were recorded. OM diagnosis was performed by HIV providers (usually attending physicians) and recorded in the patient’s medical record. Collected data were classified according to the clinical diagnostic criteria of the Clearinghouse on Oral Problems Related to HIV Infection and the World Health Organization’s (WHO) Collaborating Centre on Oral Manifestations of the Immunodeficiency virus [4]. Data were entered into a computer for analysis.

### Statistical analyses

Frequency counts and percentages were used to estimate the prevalence of OM for the descriptive statistics. Our independent variables were: age (continuous variable); gender (male vs. female), ethnicity (Bambara, Fulani, Malinke, other); marital status (single/never married, married/cohabitant, divorced/separated, widowed); education (none, some, college/university/medersa); CD4 counts at ART initiation (0-199, =200); and WHO stage (Stage I, stage II, stage III, and stage IV) at ART initiation. Categorical variables were compared using a X^2^ test. Fisher′s exact test was used when the expected values in any of the cells of the contingency table were below 5. Continuous data were compared using 2-sided t tests. The relationship between the prevalence of OM during the first month of ART and demographic and clinical variables, along with CD4 counts at ART initiation was evaluated using multiple logistic regression models. The Statistical Package for Social Sciences Software (SPSS 18 for Windows; SPSS Inc., IL, USA) was used for all of the statistical analyzes.

### Ethical review

The Ethic Committee of the Université de Bamako and the Institutional Review Board of the University of Alabama at Birmingham approved the protocol for this cross-sectional study.

## Results

The demographic, clinical and laboratory characteristics of our sample are summarized in Table 1. Our study sample consisted in 205 HIV-infected patients initiating care at Gabriel Touré Hospital with an average age of 39 years, most of them (83.0%) living in Bamako. One third of the sample (35.1%) self-identified as Bambara. Almost three-quarters of the patients (71.0%) were females and almost two-thirds (63.0%) were married. Most (90%) had very little or no education. In terms of clinical conditions at the time of HIV care initiation, almost 60% of the entire study population was classified as WHO stages III or IV (Figure1). Almost one-third (31.4%) of our study sample presented with one or more OM during the first month of ART. Most of these lesions were recorded as oral candidiasis (95%).

**Table 1:** Clinical and socio-demographic factors within a sample of HIV Malian patients who initiated ART between December 2001 and December 2008 (n=205*)

Characteristics	All patients n (%)
Age in Years (mean±SD)	39±10
**Gender**	
Female	142 (71.0)
Male	58 (29.0)
**Patient residence**	
Bamako	165 (82.9)
Other	34 (17.1)
**Ethnic Group**	
Bambara	67 (35.1)
Fulani	24 (12.6)
Senoufo	2 (1.0)
Songhay	5 (2.6)
Malinke	24 (12.6)
Dogon	9 (4.7)
Other	60 (31.4)
**Marital Status**	
Single/never married	19 (9.9)
Married/cohabitant	121 (63.0)
Divorced/separated	9 (4.7)
Widowed	43 (22.4)
**Educational Level**	
None	57 (36.1)
Some	84 (53.2)
College/University	11 (7.0)
*Medersa* (Koranik Schooling)	6 (3.8)
**CD4 (cells/ul) at ART initiation**	
0 to 199	111 (56.6)
≥ 200	85 (43.4)
**WHO Stage at ART initiation**	
WHO I	37 (19.8)
WHO II	43 (23.0)
WHO III	76 (40.6)
WHO IV	31 (16.6)
**Oral Manifestations at ART initiation**	

Absent	129 (68.6)
Present	59 (31.4)

* Due to missing data not all the variables add up to the total sample (n=205)

Patients with OM during the first month of ART were more likely to have lower CD4 counts (p<0.01) and be classified either as WHO stage III or IV at ART initiation (Table 2). The association between the OM presence/absence during the first month of ART and the other co-variates (e.g.: age, precedence, ethnicity, marital status, educational level) proved not to be statistically significant.

**Table 2:** Clinical and lab features statistically associated with presence/absence of oral manifestations among HIV Malian patients initiating ART (n=205*)

Characteristics	All patients n (%)	Without OM n (%)	With OM n (%)	*p-value*
**CD 4 Counts at ART initiation**				
0 to 199	105 (58.0)	64 (51.2)	41 (73.2)	<0.01
≥200	76 (42.0)	61 (48.8)	15 (26.8)	
**WHO Stage at ART initiation**				
WHO I	34 (19.3)	33 (27.7)	1 (1.8)	<0.01
WHO II	42 (23.9)	36 (26.9)	10 (17.5)	
WHO III	70 (39.8)	38 (31.9)	32 (56.1)	
WHO IV	30 (17.0)	16 (13.4)	14 (24.6)	
**Total number of patients**	**205 (100.0)**	**129 (68.6)**	**59 (31.4)**	

* Due to missing data not all the variables add up to the total sample (n=205); OM: Oral manifestations

For the multivariate analysis, the first logistic regression model that we ran for OM during the first month of ART (Table 3) only retained WHO Staging at ART initiation as an important covariate. CD4 count at ART initiation lost its statistical significant when the model was feed with all the co-variates. It is important to highlight that patients being classified as WHO III and WHO IV at ART initiation were 31 times more likely to develop any OM during the initial month of ART than patients in WHO stage I. However, because the wide range of our confident intervals (perhaps due to a small sample for our main outcome, OM) missing data in variable education, and low number of counts in the WHO Stage I with OM, we decided to run a separate model.For this second model, we combined stages WHO I and II, and WHO III and IV. Also, we excluded education as a co-variate (because of the high number of missing data for this variable). In the second model (Table 4), WHO staging at ART initiation was again retained as an important predictor. HIV-infected patients entering care and classified on WHO stage III or WHO stage IV at ART initiation were five times more likely to develop any OM during the first month of ART than patients with WHO stage I or WHO stage II, even after controlling for several confounders.

**Table 3:** First Model - Clinical determinants of oral manifestations^1^ during the first month of ART in the HIV cohort

	Bivariate analyses	Multivariate analyses		
	
**Predictors**	Crude OR^2^	Adj OR^2^	95% CI^3^	*p* -value
**WHO Stage**				
WHO I (ref)				
WHO II	10.31	2.965	0.215-40.909	p=0.42
WHO III	27.79	31.973	3.312-308.673	p<0.01
WHO IV	28.88	31.594	2.749-363.127	p<0.05

^1^MODEL: OM *vs*. No-OM during the first month of ART. Variables included in the model: age, ethnicity, gender, marital status, education, CD4 counts at ART initiation, WHO Stage at ART initiation

^2^Odd ratios, significant association (*p*<0.05)

^3^Confidence Interval

**Table 4:** Second Model - Clinical determinants of oral manifestations^1^ during the first month of ART in the HIV cohort

	Bivariate analyses	Multivariate analyses		
	
Predictors	Crude OR^2^	Adj OR^2^	95% CI^3^	*p*-value
WHO Stage				
WHO I and II (ref)				
WHO III and IV	5.034	5.400	2.400-12.149	p<0.01

^1^MODEL: OM *vs*. No-OM during the first month of ART. Variables included in the model: age, ethnicity, gender, marital status, CD4 counts at ART initiation, WHO Stage at ART initiation.

^2^Odd ratios, significant association (*p*<0.05);

^3^Confidence Interval

## Discussion

HIV-related OM have been widely studied in developed nations [11,12]. However, few epidemiological reports from the African continent are available, especially from West Africa. This study has highlighted the prevalence of OM in HIV-infected patients on ART starting care at the Gabriel Touré Hospital. Consistently with other Malian and West-African reports [3,8–10], our sample consisted in mostly women (71%) with 63% of the subjects being married, and 39 years as median age at ART initiation. Local customs may promote unsafe sexual behaviors in Mali, by encouraging men to have many sexual partners and older men to have sexual relations with much younger women, thereby contributing to the higher infection rates among Malian women [5], Also the median age of our study population could be highlighting the fact that most of these patients are probably experiencing delayed entry into HIV care.

Our results showed that 31.4% of the HIV-infected patients presented at least one OM during the first month of ART. This prevalence rate is lower than the ones reported by other African countries like Nigeria (84.0%) [13], Lesotho (73.0%) [14], Uganda (72.0%) [15], and South Africa (60.0%) [16]. However, we believe that OM may be under-reported or misclassified due the lack of dental care providers performing the intra-oral evaluation of the HIV patients at the Gabriel Touré Hospital. Therefore, oral/dental training for HIV care providers, including physicians and nurses among others, should be a priority in this type of setting.

Similarly to the studies mentioned above, oral candidiasis was the most prevalent OM in our study sample. This constituted an important management challenge since it has been demonstrated that HIV-infected patients with presenting oral candidiasis are at increased risk for progression to AIDS [17,18].

Almost 60% of the entire sample was on WHO stage III or WHO stage IV, and 56756.6% had low CD4 counts (less than 200 cells/µl) at ART initiation. This indicates that HIV patients experience delayed entry into care, along with important immuno-suppression and with clinical co-morbidities. This is most likely the result of HIV stigmatization [6,13], and lack of access/utilization of available health facilities, especially among women [19,20]. The fact that HIV-infected patients entering care and classified on WHO stage III or WHO stage IV were five times more likely to develop an OM during the first month of ART must be taken into consideration for future research programs, protocols design and HIV care management, especially among patients initiating antiretroviral regimens. This will be important for optimizing pre-ART and ART management especially in resource-limited countries such as Mali.

The data used for this study, therefore our findings should be interpreted with caution due the following limitations: 1) data were secondary data, (not originally collected for the purpose of this study), which means that OM may have been under-reported or misclassified by providers and this could lead to an underestimation of prevalence. Missing data and small sample size presented daunting challenges for this study therefore our findings should be interpreted with caution. The small sample size for multivariate analyses due to small counts in some categories of the explanatory variables lead us to being unable to assess important risk factors for OM, especially tobacco and alcohol use, which are known to be associated with presence/absence of OM. Additionally, our data collection instrument has not been validated by other studies.

## Conclusion

Based on the findings of our study, we still believe that strategies for dental/oral training of health professionals are required in order to prevent pain, discomfort, malnutrition, weight loss, dehydration, dry mouth, non-adherence to ART, and ultimately death. Including oral health in the package of care for the HIV patients in Mali should be considered as an urgent priority.

## Additional material

**Additional material 1:** Data Abstraction Tool
